# Polyethylene terephthalate nanoplastics-induced neurotoxicity in adult male Swiss albino mice with amelioration of betaine: a histopathological, neurochemical, and molecular investigation

**DOI:** 10.1007/s00210-025-03867-9

**Published:** 2025-02-12

**Authors:** Nehal A. Kamel, Dina W. Bashir, Ebtihal M. M. El-Leithy, Adel F. Tohamy, Maha M. Rashad, Ghada E. Ali, Abdel Aleem A. El-Saba

**Affiliations:** 1 Department of Cytology and Histology, Faculty of Veterinary Medicine, Cairo University, Giza, 12211 Egypt; 2 Department of Toxicology and Forensic Medicine, Faculty of Veterinary Medicine, Cairo University, Giza, 12211 Egypt; 3 Department of Biochemistry and Molecular Biology, Faculty of Veterinary Medicine, Cairo University, Giza, 12211 Egypt

**Keywords:** Polyethylene terephthalate nanoplastics, Neurotoxicity, Betaine, Neurochemical markers

## Abstract

Medicines, food packaging, personal care products, and cosmetics extensively use polyethylene terephthalate nanoplastics (PET-NaPs). However, they also have harmful impacts on several organs. Betaine demonstrates potent antioxidant and anti-inflammatory characteristics. Our goal was to investigate the detrimental impact of PET-NaPs on the mouse brain and evaluate the neuroprotective properties of betaine. We allocated 40 completely mature male Swiss albino mice into four distinct groups: control group, betaine group, PET-NaPs group, and betaine-co-treated group. Following a 30-day duration, euthanasia was performed on the mice, and analyzed tissue samples were obtained from the cerebrum, cerebellum, and hippocampus. PET-NaPs resulted in an elevated level of malondialdehyde and upregulated cyclooxygenase-2 and interleukin-1 beta (IL-1β) expression while significantly reducing the levels of glutathione and downregulating acetylcholinesterase. The PET-NPs also caused significant changes in the histopathology of the brain tissue, and there was a demonstrable rise in the immunostaining of IL-1β and glial fibrillary acidic proteins. Consequently, betaine effectively alleviated the negative consequences of PET-NaPs. Therefore, betaine possesses the capacity to mitigate the neurotoxic consequences induced by PET-NaPs.

## Introduction

Nanotechnology and nanoengineering possess the capacity to generate significant scientific and technical advancements in the fields of medicine and physiology. Nanotechnology is a field of science and engineering that focuses on designing, synthesizing, characterizing, and using materials and devices with minimal functional organization on the nanoscale scale, typically ranging from a few to several hundred nanometers. A nanometer equals one billionth of a meter, three orders of magnitude smaller than a micron (Silva 2004). Biodetection of pathogenic organisms (Edelstein et al. 2000), fluorescent biological labels (Wang et al. 2002), medication and genetic delivery (Pantarotto et al. 2003), detecting proteins (Nam et al. 2003), probing of DNA structure, the engineering of tissues (Ma et al. 2003), and cancer deterioration via heating (hyperthermia) are all applications of nanomaterials in medicine and science. Previous research has shown that increased human exposure to nanoparticles leads to a wide range of health risks (Sadiq et al. 2011). Nanoplastics (NaPs), described as plastic debris with a diameter of 100 nm (Gewert et al. 2015; Gigault et al. 2016), gather in the surrounding environment as a consequence of plastic deterioration by ultraviolet (UV) sunlight and microbial organisms (Yousif and Haddad 2013; Song et al. 2017; Wilkinson et al. 2017), and release directly from electronic items, food containers (Eleftheriadou et al. 2017), personal care products, and fabrics (Contado 2015). Melt-phase condensation and solid-state polymerization combine terephthalic acid from petroleum and ethylene glycol to synthesize polyethylene terephthalate (PET). PET is widely used to produce plastic bottles and caps. Researchers have detected it in pharmaceuticals, food packaging, and beverages, including carbonated drinks. Researchers have discovered microplastics (MPs) in beer (Shruti et al. 2020a) and mineral waters stored in glass bottles (Mammo et al. 2020), presumably due to bottle degradation. The worldwide production of PET resin in 2020 amounted to 30.1 million metric tons, as Rakesh et al. (2021) reported. According to the American eating pattern, adults can consume 5 g of MPs per week, as Clere et al. (2022) stated. Additionally, the recommended daily consumption for PET-MPs is 166 mg, according to Tamargo et al. (2022). However, there is currently no available data about humans’ consumption of polyethylene terephthalate nanoplastics (PET-NPs). Schwabl et al. (2019) conducted a prospective pilot experiment that revealed the presence of PET particles in human feces, suggesting that these particles actively interact with the human digestive system. NPs can get into people’s bodies through the food chain (Van Cauwenberghe and Janssen 2014), consumption of water (Shruti et al. 2020b), inhaling (Chen et al. 2020), and ingestion of personal care items (Chen et al. 2020). Inhalation and oral exposure are primary entry routes (Zarus et al. 2021). Prior studies have demonstrated that the central nervous system (CNS) is a favorable objective for MPs (Hu and Palić, 2020), and NPs pose a greater risk than MPs due to their propensity to accumulate in the brain and traverse the blood–brain barrier (BBB) more readily. Furthermore, studies have found that this substance can lead to developmental neurotoxicity and hinder locomotor activity in zebrafish larvae (Chen et al. 2017); it also causes behavioral alterations, including swimming inadequacy (Pitt et al. 2018), which can reduce exploratory activity (Mattsson et al. 2017), as well as inflammation, genotoxicity, and even carcinogenicity (Xu et al. 2019; Poma et al. 2019; Hu et al. 2021). Researchers have reported that nanoparticles (NPs) cause harmful effects by producing reactive oxygen species (ROS), leading to oxidative stress and the subsequent synthesis of free radicals (Barboza et al. 2018; Pitt et al. 2018; Liu et al. 2019). We have not yet studied the neurotoxicity of PET-NaPs. Therefore, addressing this deficiency will have implications for the cautious utilization of these particles.

Adding a methyl group to the amino acid glycine forms the element betaine. Organs such as the liver endogenously produce this substance (Preedy 2015; Day and Kempson 2016), and fish, beans, and grains also contain significant amounts (Zeisel et al. 2003; Ross et al. 2014). This substance quickly absorbs and functions as an osmolyte and methyl donor due to the three methyl groups in its composition (Kempson and Montrose 2004; Olthof and Verhoef 2005). Multiple research investigations have shown that betaine protects the brain against various poisons (Söğüt and Kanbak 2016). Betaine has many pharmacological effects on neurons, including antidepressant, antioxidant, anti-seizure, and memory-enhancing qualities (Rowley 2011; Chai et al. 2013; Kim et al. 2013; Kunisawa et al. 2015, 2017; Lin et al. 2016; Nie et al. 2016). It is intriguing to note that betaine can decrease neuroglial activation, which in turn helps protect mice against neuroinflammation generated by lipopolysaccharide (LPS) (Miwa et al. 2011; Amiraslani et al. 2012). The potential of betaine to protect against neurotoxicity is a promising area of research that could have significant implications for the fields of nanotechnology, medicine, and toxicology.

Due to a lack of studies on PET-NaPs, our goal was to investigate if PET-NPs could trigger harmful effects on the cerebrum, cerebellum, and hippocampus of Swiss albino mice. Additionally, we investigated the protective effects of betaine against the neurotoxicity caused by PET-NaPs. The findings of this study could have significant implications for the cautious utilization of PET-NaPs in various applications, particularly in the fields of nanotechnology, medicine, and toxicology.

## Materials and methods

### Chemicals and reagents

Betaine (C_5_H_11_NO_2_; Amargain industrial complex, Opp. S.T. Stand, LBS, Marg, Khopat Thane Mumbai, Maharashtra, India) was obtained from El-Mekkawy Company, Cairo, Egypt.

PET-NaP preparation was performed at the National Research Centre, 33 El Bohouth St. 12622, Dokki, Giza, 12311, Egypt. All chemicals and reagents used were of analytical grade.

The chemicals that were used in this study including TFA, PET, and SDS were purchased from Sigma-Aldrich. For all experiments, deionized water (Barnstead Nanopure Ultrapure Water System) was used.

### Preparation of PET-NPs

Precipitation techniques that had been previously documented were employed to generate PET-NaPs (Kamel et al. 2024a, b).

### Characterization of PET-NPs

#### Transmission electron microscopy

Transmission electron microscopy (TEM; JEM-2100; JOEOL Co., Tokyo, Japan) was employed to identify the morphology of the synthesized PET-NaPs under high-tension electricity of 160 kV at room temperature. The photographs were taken at high magnifications to estimate the morphology and diameter of the PET-NaPs.

#### Particle size analysis of the prepared PET-NPs

Dynamic light scattering (DLS) techniques (Nicomp380ZLS, DLS equipment) were used to analyze the hydrodynamic particle size of PET-NaPs.

### Experimental animals and ethical approval

We divided 40 mature male Swiss albino mice (obtained from the VACSERA animal home in Egypt) into four groups, each containing ten mice housed in five cages. Over a 30-day period, several treatments were administered to the mice, using a gavage needle, including daily oral dosages and intraperitoneal (i.p.) injections.

The control group, known as group I, was administered distilled water. Kamel et al. (2024a) described the administration of betaine alone to group II, also known as the betaine-treated group, at a 1000-mg/kg body weight per day intraperitoneally. Group III, also known as the PET-NPs group, was administered PET-NaPs orally via a gavage needle at a dose of 200 mg/kg body weight per day, following the protocol described by Kamel et al. (2024b). Group IV received betaine at 1000 mg/kg body weight per day, followed by PET-NaPs at 200 mg/kg body weight.

The experimental methodologies were employed in the Department of Pathology, Faculty of Veterinary Medicine, Cairo University.

### Sample collection and preparation

After 30 days of treatment, mice were anesthetized with 2% isoflurane (0.10 ml isoflurane liquid in the internal volume of the chamber (1 l)) and sacrificed by cervical decapitation within 30 min. Brain samples (cerebrum, cerebellum, and hippocampus) were extracted. Cold phosphate-buffered saline (PBS; pH 7.4) was used to homogenize a portion % of the collected tissues into a 40% homogenate. These tissue homogenates were aliquoted and kept at − 80 °C until biochemical analysis. The remaining tissues were preserved in a 10% neutral-buffered formalin (NBF) solution for histopathology and immunohistochemical analysis.

### Biochemical analyses

Each gram of brain tissue was homogenized in 5 ml ice-cold potassium phosphate buffer (50 mM) containing 1 mM EDTA using a tissue homogenizer, followed by centrifugation at 4000 rpm for 15 min, and the supernatant was collected. Reduced glutathione (GSH) and malondialdehyde (MDA) levels were determined in brain tissue homogenates using colorimetric kits purchased from Bio-diagnostic Co., Giza, Egypt, following the manufacturer’s instructions.

#### Quantitative real-time PCR analysis for acetylcholinesterase (*AChE*), cyclooxygenase-2 (*COX-2*), and interleukin-1 beta (*IL-1β*) genes in the brain

The relative cerebral, cerebellar, and hippocampal *AChE*, *COX-2*, and *IL-1β* messenger RNA (mRNA) abundance were determined by quantitative real-time PCR (RT-PCR) using *GAPDH* as a housekeeping gene (Noshy et al. 2023). Approximately 50 mg of cerebral, cerebellar, and hippocampal tissues was used for total RNA extraction using the total RNA extraction kit (Vivantis, Malaysia; Cat. No. AM1924). A NanoDrop spectrophotometer was used to determine RNA concentration and purity (Hassan et al. 2023). RT-PCR was carried out using M-MuLV Reverse Transcriptase (NEB#M0253) (Bashir et al. 2021). For performing RT-PCR analysis, the SYBR Green PCR Master Mix (Thermo Scientific; Cat. No. K0221) was used (Hashim et al. 2022). Sequences of primers used are as follows: for AChE, forward: 5′-CATGCACATACTGTCCCTGC-3′, reverse: 5′-CTTT CTTGAGGCA GGACGTG-3 (Valuskova et al. 2017); for *Cox-2*, forward: CATCCC CTTCCTGCGAAGTT: reverse: CATGGGAGTTGGGCAGTCAT (Atta et al. 2023); and for *IL-1β*, forward: 5′-ACTCATTGTGGC TGTGGAGA-3′, reverse: 5′-TTGT TCATCTCG GAGCCTGT-3′. Each quantitative RT-PCR was performed with three biological replicates, and each biological replicate was assessed three times (Elmosalamy et al. 2022). In each experiment, template-free negative controls were included (Hassan et al. 2023). The comparative 2^−ΔΔCT^ method was used to calculate the relative transcription levels (Rashad et al. 2018).

### Histopathological analyses

#### Light microscopy

We preserved the brain tissues, including the cerebrum, cerebellum, and hippocampus, in a 10% NBF solution for 48 h after meticulous dissection. Subsequently, the samples underwent a series of procedures, including washing, dehydration using ethyl alcohol in increasing concentrations, clarification using xylene, and finally embedding in paraffin wax. We prepared 4-mm-thick slices using a rotatory microtome. These sections were then deparaffinized and stained with hematoxylin and eosin (H&E) for observation under a light microscope (Bancroft et al. 2013).

##### Histopathological scoring

We classified and scored the brain damage under a microscope in a blinded manner, following the methods described by Hassanen et al. (2021). We assessed the histopathological lesions and assigned a grade and score ranging from 0 to 4. The grading system is as follows: 0 indicates normal histology without any alterations; 1 indicates light tissue damage, which is 0.5% or less; 2 indicates moderate tissue damage, which is between 25 and 50%; 3 indicates severe tissue damage, which is between 50 and 75%; and 4 indicates extensive severe tissue damage, which is more than 75%. We quantified lesion severity in three microscopic fields per six slices, representing six animals in each group.

##### Immunohistochemical analysis of glial fibrillar acid protein (GFAP)

The procedure delineated in the work of Stoltenburg-Didinger et al. (1996) involved detecting astrocyte proteins in the hippocampus, cerebellum, and cerebrum using paraffin sections on glass transparencies coated with poly-l-lysine. Sections were treated with xylene and acetone for 10 min, followed by phosphate-buffered saline, methanol, and H_2_O_2_ for 30 min to eliminate peroxidase activity. Two PBS rinses were used between immune reagent changes, followed by incubation in normal goat serum for 20 min to prevent contamination. Samples were incubated with polyclonal rabbit anti-GFAP antiserum in PBS, followed by adding biotinylated mouse anti-rabbit immunoglobulin and incubating for 30 min to form an avidin–biotin complex.

##### Immunohistochemical analysis of *IL-1β *

Brains were extracted and sliced (3–4 µm thick) with a sliding microtome. Following an hour of blocking in a solution comprising 10% normal goat serum diluted in Tris-buffered saline (TBS; pH 7.4) with 0.2% Triton X-100 (TBS-Tx 0.2%), free-floating sections were incubated overnight at 4 °C in the same buffer solution with primary antibodies against IL-1β (1:100). The sections were rinsed, incubated with a biotinylated anti-rabbit secondary antibody, linked to horseradish peroxidase, and observed using diaminobenzidine (DAB) histochemistry. DAB exposure time was comparable for control and experimental samples. Finally, slices were washed entirely in TBS before mounting on gelatin-coated slides, air-drying, dehydrating in ethanol, clearing in xylenes, and mounting with cytosol (Stephens Scientific, Wayne, NJ, USA) (Fuentes-Santamaría et al. 2013).

#### Evaluation of immunohistochemical observations (area percent)

We evaluated the sections that underwent immunohistochemical staining using ImageJ. This study used a standard measuring frame and a × 400 light microscope to find the percentage of immunohistochemical responses in five fields from different transparencies within each group. We then displayed the results on the monitor’s screen. Regardless of the staining strength, we chose regions demonstrating a favorable immunohistochemical response for investigation. We rendered these areas indistinct in the context of computer system measurement using a blue binary hue. We computed each specimen’s mean value and standard error (SE), followed by a statistical analysis.

### Statistical analysis

The findings were reported as the mean ± SE using SPSS program version 27. To ensure the validity of the results, the data were meticulously analyzed via one-way analysis of variance (ANOVA), which was followed by the LSD post hoc test. A *P* value below 0.05 was deemed to indicate statistical significance.

## Results

### Characterization of PET-NaPs

#### Determination of PET-NaP morphology by TEM

**Fig. 1:**
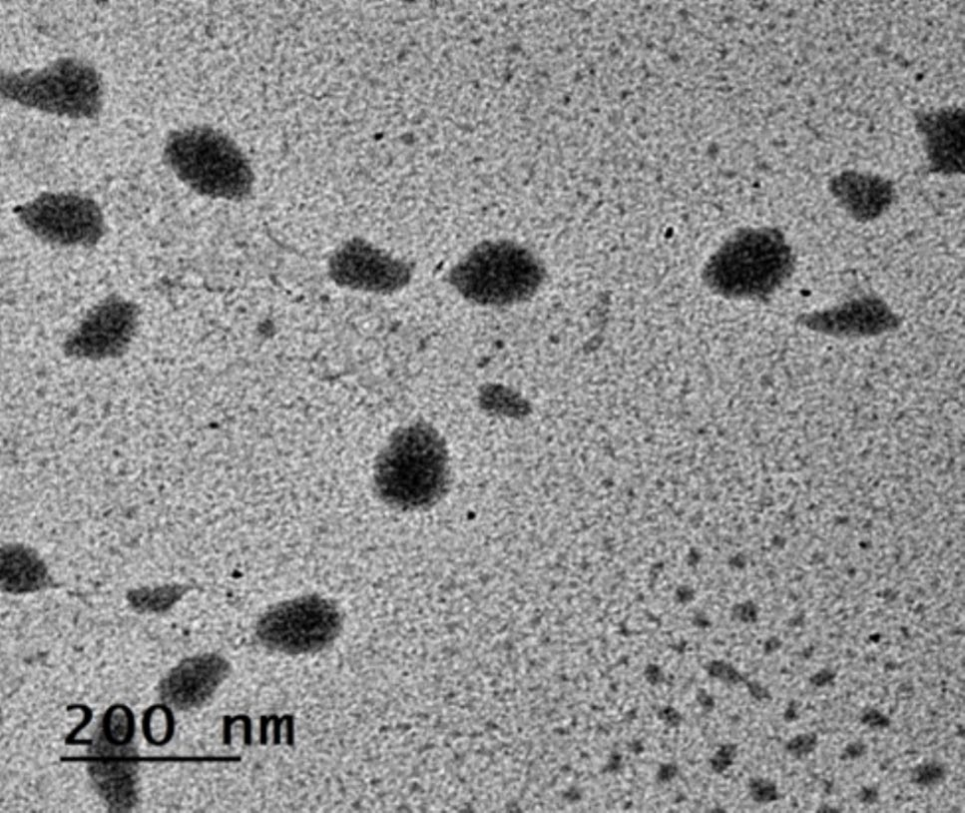
PET-NaPs by TEM

TEM at high magnifications examined the synthesized PET-NPs to observe the particle morphology, as shown in Fig. 1. Polyethylene terephthalate nanoparticles (PET-NaPs) have a symmetrical morphology, characterized by an average diameter of around 83 nm. In addition, TEM pictures show that the PET-NPs do not form particle aggregates or agglomerates. Instead, they consist of uniformly distributed particles of the same size.

#### Determination of PET-NaP size

**Fig. 2:**
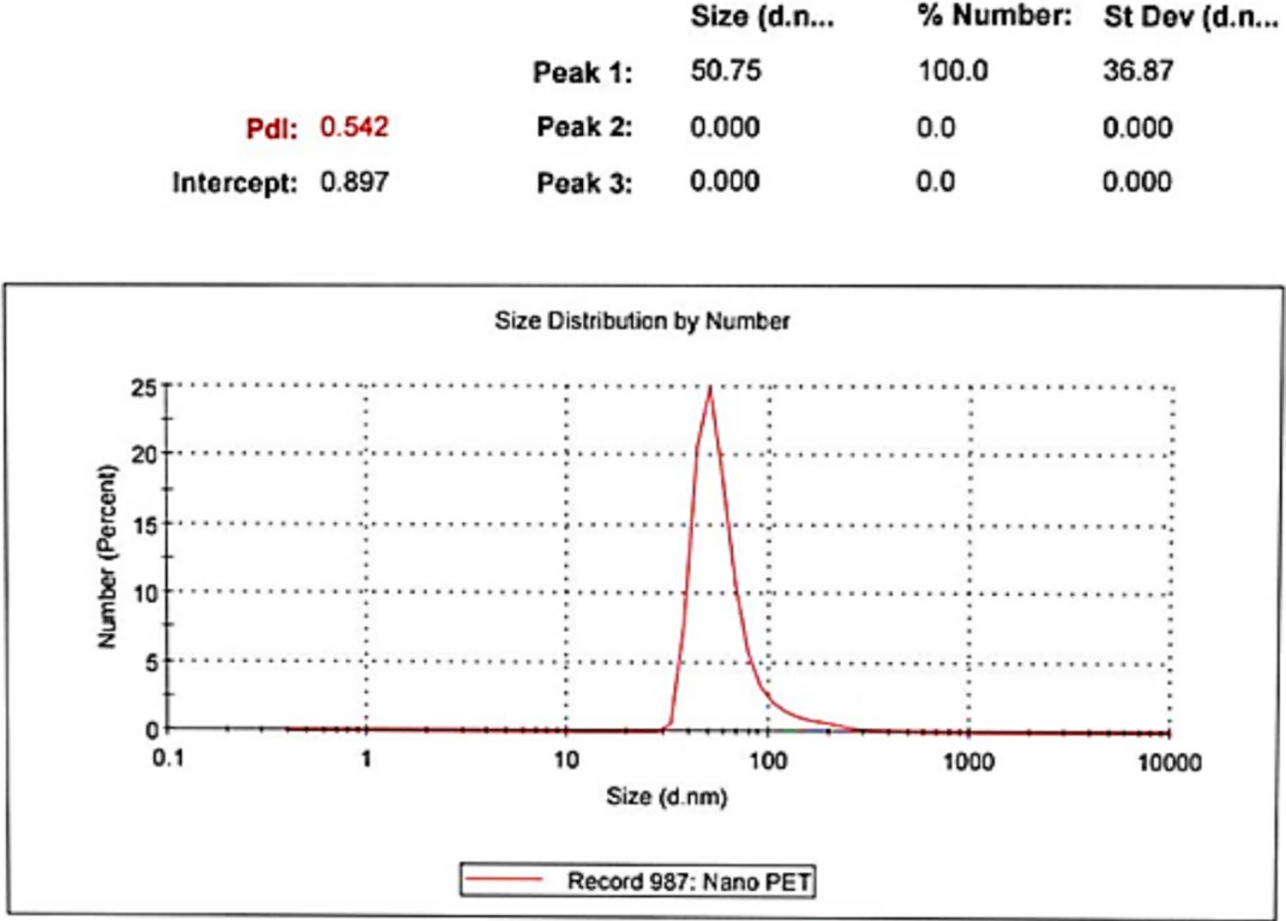
Particle size of PET-NPs by DLS

DLS was also utilized to measure the average hydrodynamic size of the synthesized polystyrene nanoplastics (PS-NaPs), which revealed that they have an average size of 50.75 nm and a polydispersity index (PDI) of 0.542 (Fig. 2).

### Biochemical investigations

#### Oxidative stress biomarkers

**Fig. 3:**
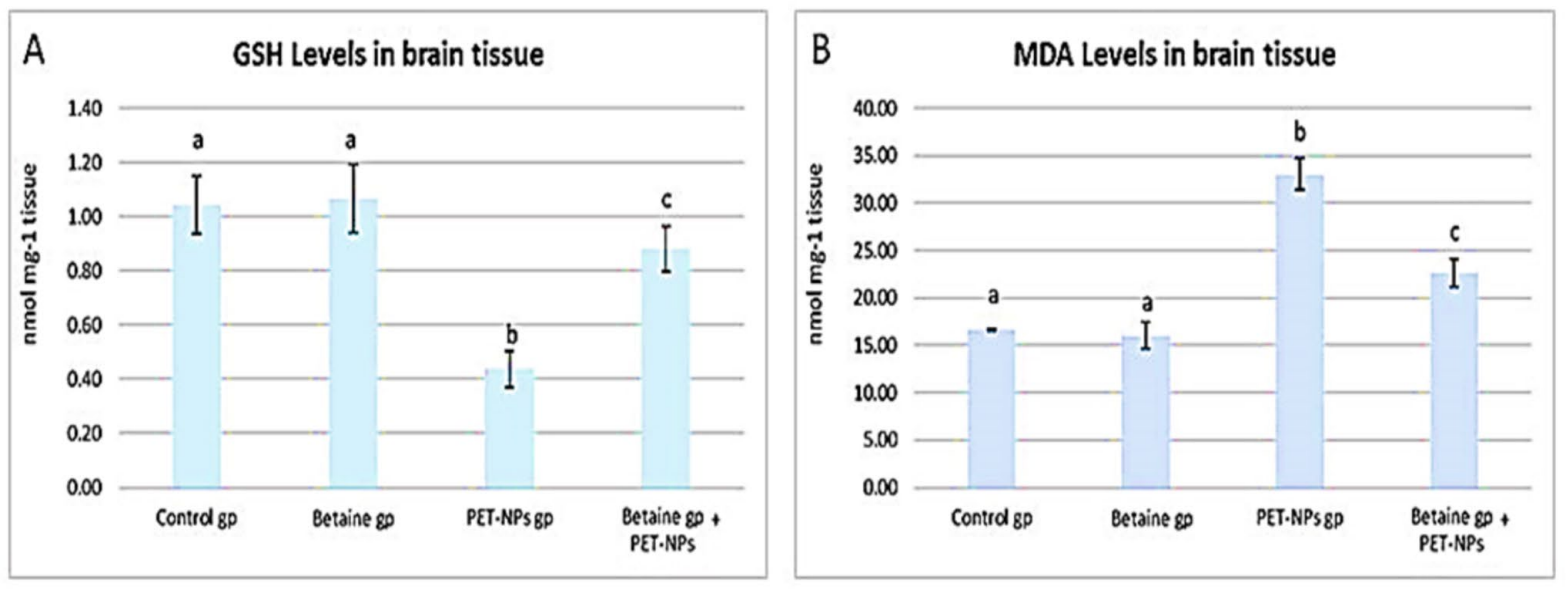
Effects of PET-NPs and betaine on the levels of GSH (**A**) and MDA (**B**) (nmol/mg tissue) in male mice (*n* = 5 mice/group). Data are represented as mean ± SEM. Groups having different letters are significantly different from each other at *P* ≤ 0.05. Groups with similar letters are non-significantly different at *P* ≤ 0.05

We used the levels of GSH and MDA to detect oxidative stress indicators in brain tissue. The administration of PET-NaPs resulted in oxidative stress in the brain tissue, as evidenced by a substantial reduction in GSH levels and an increase in MDA levels compared to the control group (*P* ≤ 0.05). Figure 3 shows that adding betaine improved the oxidative stress caused by PET-NaPs. This was achieved by significantly increasing the levels of GSH and decreasing the levels of MDA compared to the group treated with PET-NaPs alone (*P* ≤ 0.05).

#### Quantitative real-time PCR

##### mRNA relative expression of ***AChE*** gene 

**Fig. 4:**
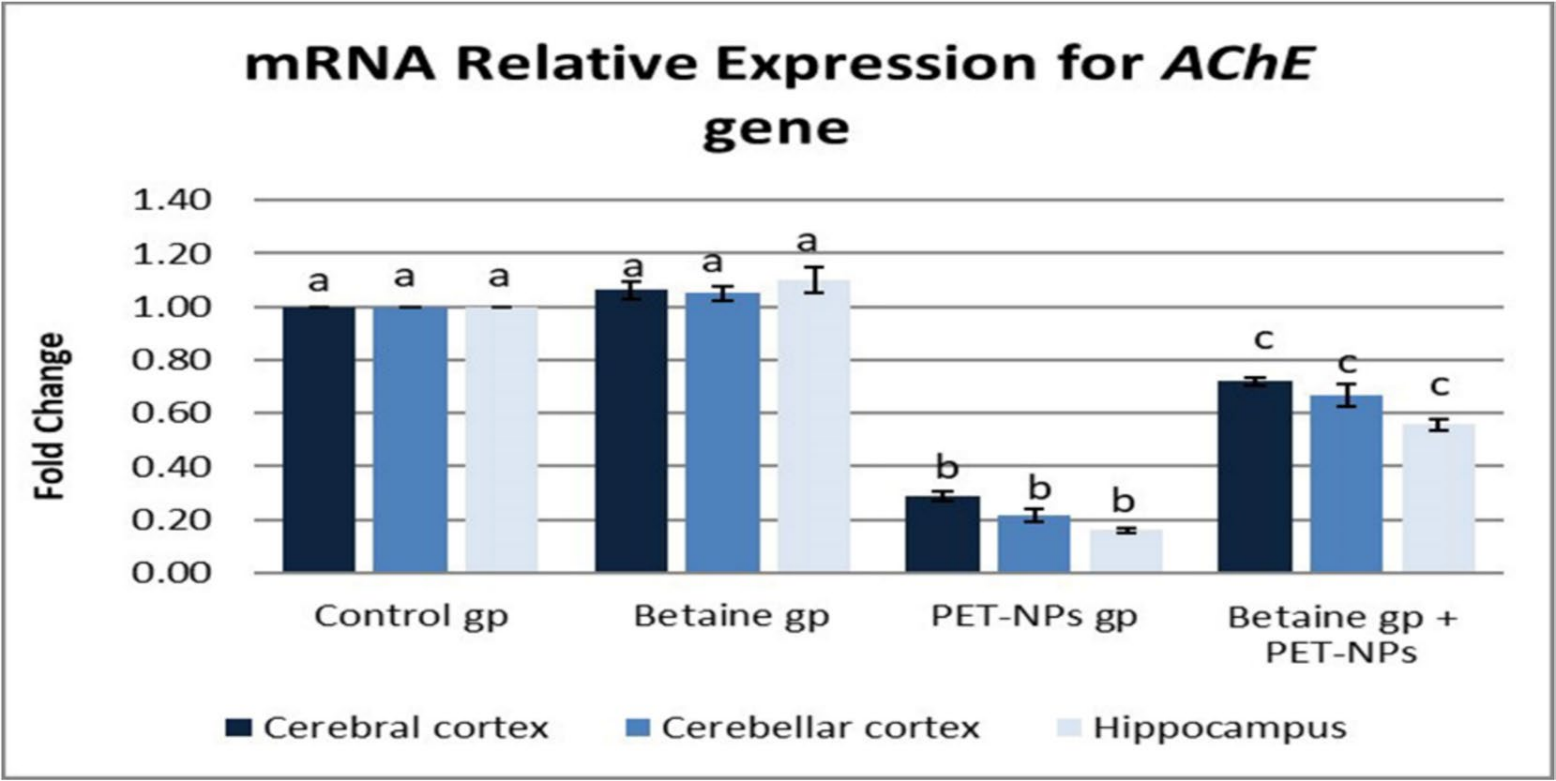
Effects of PET-NaPs and betaine on mRNA relative expression of the *AChE* gene in male mice (*n* = 5 mice/group). Data are represented as mean ± SEM. Groups having different letters are significantly different from each other at *P* ≤ 0.05. Groups with similar letters are non-significantly different at *P* ≤ 0.05

The administration of PET-NaPs resulted in a significant decrease in AChE expression in the cerebrum, cerebellum, and hippocampus. The downregulation was 0.29-fold, 0.22-fold, and 0.16-fold, respectively, compared to the control group (*P* ≤ 0.05). As shown in Fig. 4, giving betaine simultaneously increased the expression of AChE in the cerebrum, cerebellum, and hippocampus.

##### mRNA relative expression of some inflammation-related genes (***COX-2*** and ***IL-1β***) 

**Fig. 5:**
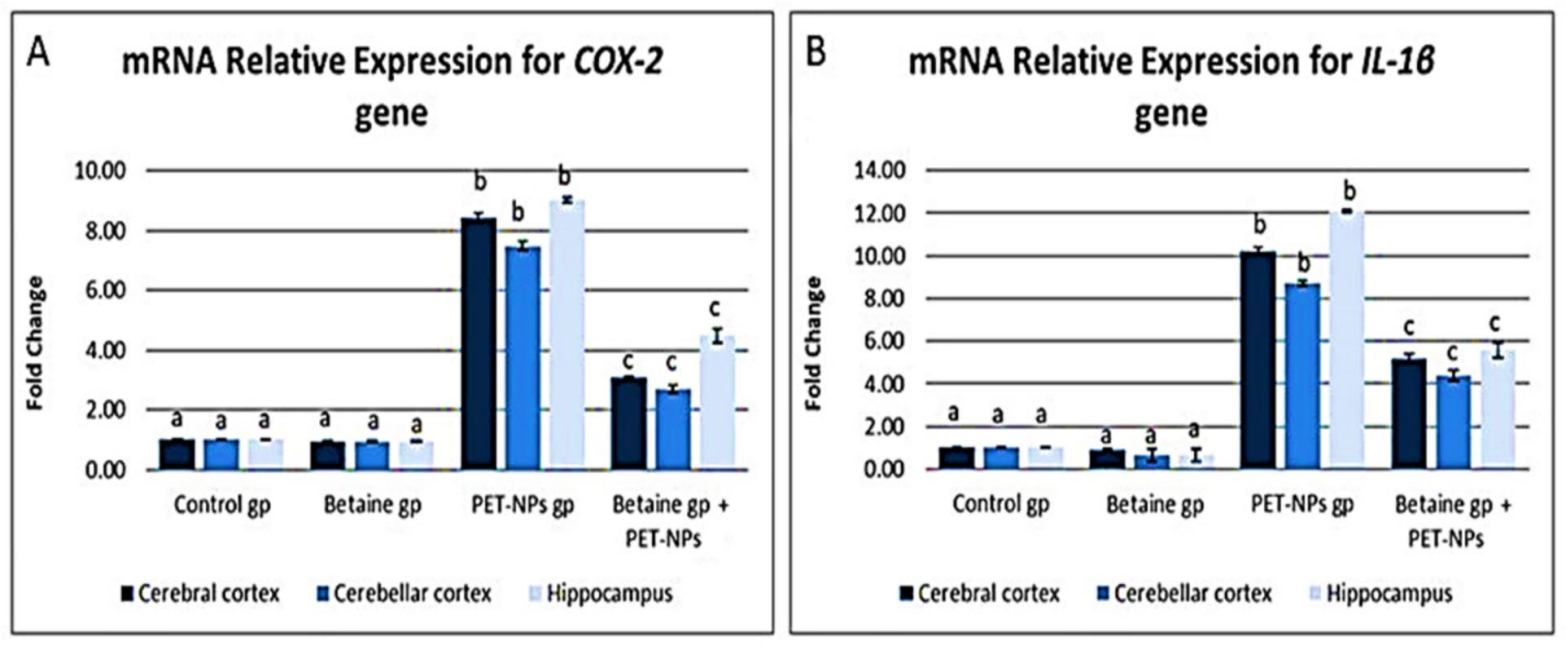
Effects of PET-NaPs and betaine on mRNA relative expression of some inflammation-related genes (**A** *COX-2* and **B** *IL-1β*) in male mice (*n* = 5 mice/group). Data are represented as mean ± SEM. Groups having different letters are significantly different from each other at *P* ≤ 0.05. Groups with similar letters are non-significantly different at *P* ≤ 0.05

Findings in Fig. 5A demonstrated that PET-NaPs significantly increased the expression of the *COX-2* gene. The fold increase in expression was 8.43, 7.47, and 9.03 in the cerebrum, cerebellum, and hippocampus, respectively, compared to the control group (*P* ≤ 0.05). The addition of betaine resulted in a considerable decrease in the mRNA expression of the *COX-2* gene in the cerebral, cerebellar, and hippocampal tissues.

The expression of *IL-1β* was considerably increased in the group treated with PET-NaPs. The increase was 10.25-fold in cerebral tissue, 8.70-fold in cerebellar tissue, and 12.11-fold in hippocampus tissue, compared to the control group (*P* ≤ 0.05). As shown in Fig. 5B, there was an apparent decrease in the expression of this gene in the cerebral, cerebellar, and hippocampal tissues between the groups treated with PET-NaPs and those treated with both PET-NaPs and betaine.

### Histopathological investigation

#### Light microscopic observations

**Fig. 6:**
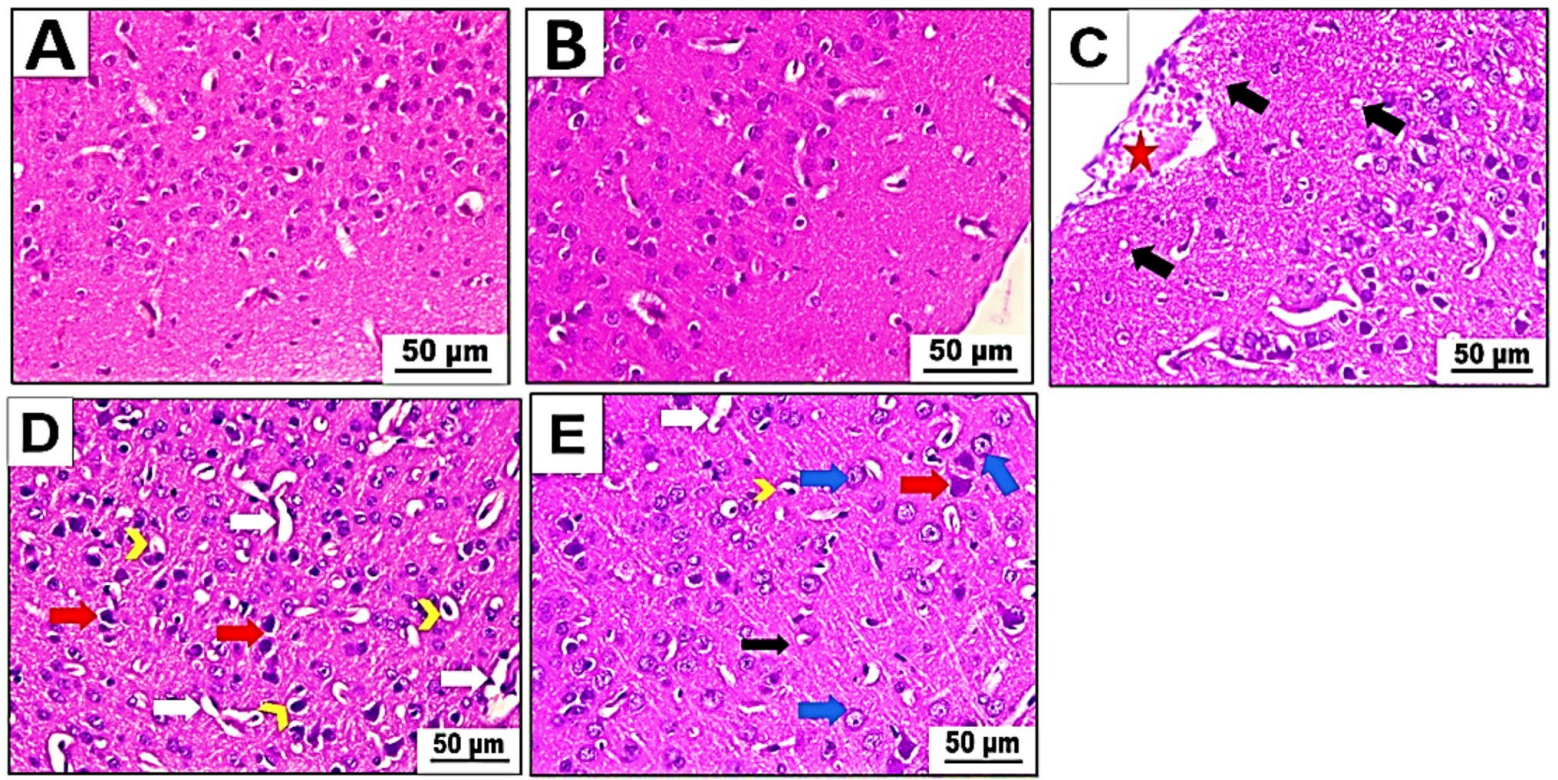
**A**–**E** Photomicrographs of cerebral cortex sections of Swiss albino mice (*n* = 5 mice/group). H&E stain (× 400). **A** Control group and **B** betaine-exposed group showing standard structure and distribution of neurons, neuroglia, and neuropil. **C**, **D** PET-NPs-exposed group. **C** Revealing subpial hemorrhage (star) and vacuolation of neuropil (black arrow). **D** Cerebral cortex displaying pericellular spaces (arrowhead), perivascular spaces (white arrow), and degenerated neurons with pyknotic nuclei (red arrow). **E** Betaine-co-treated group demonstrating partial recovery in terms of diminished neuropil vacuolations (black arrow), pericellular (arrowhead) and perivascular spaces (white arrow), almost-normal neurons (blue arrow), and just a few neurons showing degeneration and pyknosis (red arrow)

The brain slices from two groups of mice—the control group (group I) and the betaine-administered group (group II)—showed a standard histological structure when stained with H&E. The sections displayed intact normal neurons, neuroglia cells, and neuropil. The neurons are round and centrally located and have vesicular nuclei with pale basophilic cytoplasm (Fig. 6A, B). On the other hand, when we looked at brain slices from mice that had been given PET-NaPs (group III), we saw subpial bleeding and vacuolation of the neuropil (spongiosis) (Fig. 6C). In addition, the spaces between cells and blood vessels have grown, the organization of neurons in the brain’s layers has been messed up, and there are deformed, dying, and smaller neurons with darkly stained nuclei (Fig. 6D). We studied sections of the cerebral cortex from mice (group IV) treated with both betaine and PET-NaPs. The results revealed that betaine positively impacted the cerebral cortex affected by PET-NaPs. The reduced presence of neuropil vacuolations, pericellular spaces, and perivascular spaces indicated partial recovery. In addition, most neurons seemed almost expected, exhibiting basophilic cytoplasm and weakly stained nuclei. However, a few neurons exhibited degeneration and pyknosis, as seen in Fig. 6E.

**Fig. 7:**
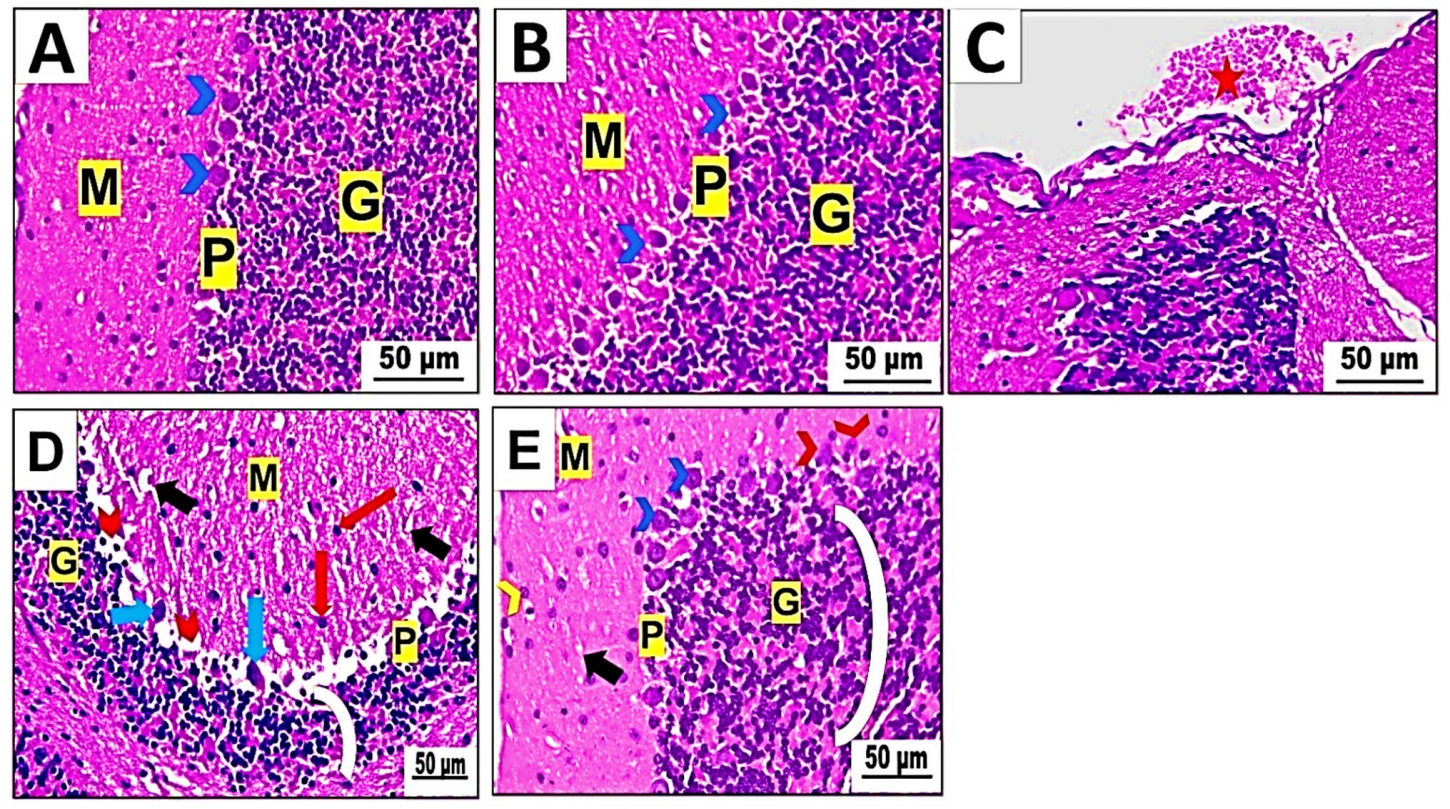
**A**–**E** Photomicrographs of cerebellar cortex sections of Swiss albino mice (*n* = 5 mice/group). H&E stain (× 400). **A** Control group and **B** treated group showing a standard molecular layer (M), a Purkinje cell layer (P) with standard-shaped Purkinje cells (arrowhead), and a granular layer (G). **C**, **D** PET-NaPs-exposed group revealing hemorrhage in the pia matter (star) (**C**) and displaying nuclear pyknosis (red arrow) and vacuolation in neuropil (black arrow) in M, degeneration of Purkinje cell in P with pyknotic nuclei (blue arrow), and pericellular spaces (arrowhead), and a decrease in thickness of G (arc) (**D**). **E** Betaine-co-treated group showing evidence of restoring, such as decreased neuropil vacuolation of M (black arrow) and nuclear pyknosis (yellow arrowhead). P seems virtually normal with normal Purkinje cells (blue arrowhead), except some are still shrunken (red arrowhead) with an increase in G thickness (arc)

From what we saw, the cerebellar cortex of two groups of mice—the control group (group I) and the betaine-exposed group (group II)—had a typical structure with three layers. There is a molecular layer on the outside with small stellate neurons, a middle layer with normal flask-shaped Purkinje cells that have lightly stained nuclei and basophilic cytoplasm, and a granular layer on the inside with neurons that are darkly stained (Fig. 7A, B). In contrast, our research reveals the effects of PET-NP delivery on the cerebellar cortex compared to the control group. Light microscopy of mice treated with PET-NaPs (group III) showed much bleeding in the pia matter (Fig. 7C), empty spaces in the neuropil, and nuclear pyknosis in the molecular cell layer. The Purkinje cell layer was also looked at, and distorted and smaller Purkinje cells with squished nuclei were found, along with the complete absence of multiple Purkinje cells.

Furthermore, pericellular gaps surround specific Purkinje cells. The granular cell layer reduced thickness, as seen in Fig. 7D. Group IV showed that betaine improved the effects of PET-NaPs on the cerebellar cortex. In the molecular layer, there was a notable decrease in neuropil vacuolation and nuclear pyknosis. Most Purkinje cells showed normal morphology and had vesicular nuclei, with decreased gaps surrounding the nerves in the Purkinje cell layer. Furthermore, the granular cell layer had an almost typical thickness (Fig. 7E).

**Fig. 8:**
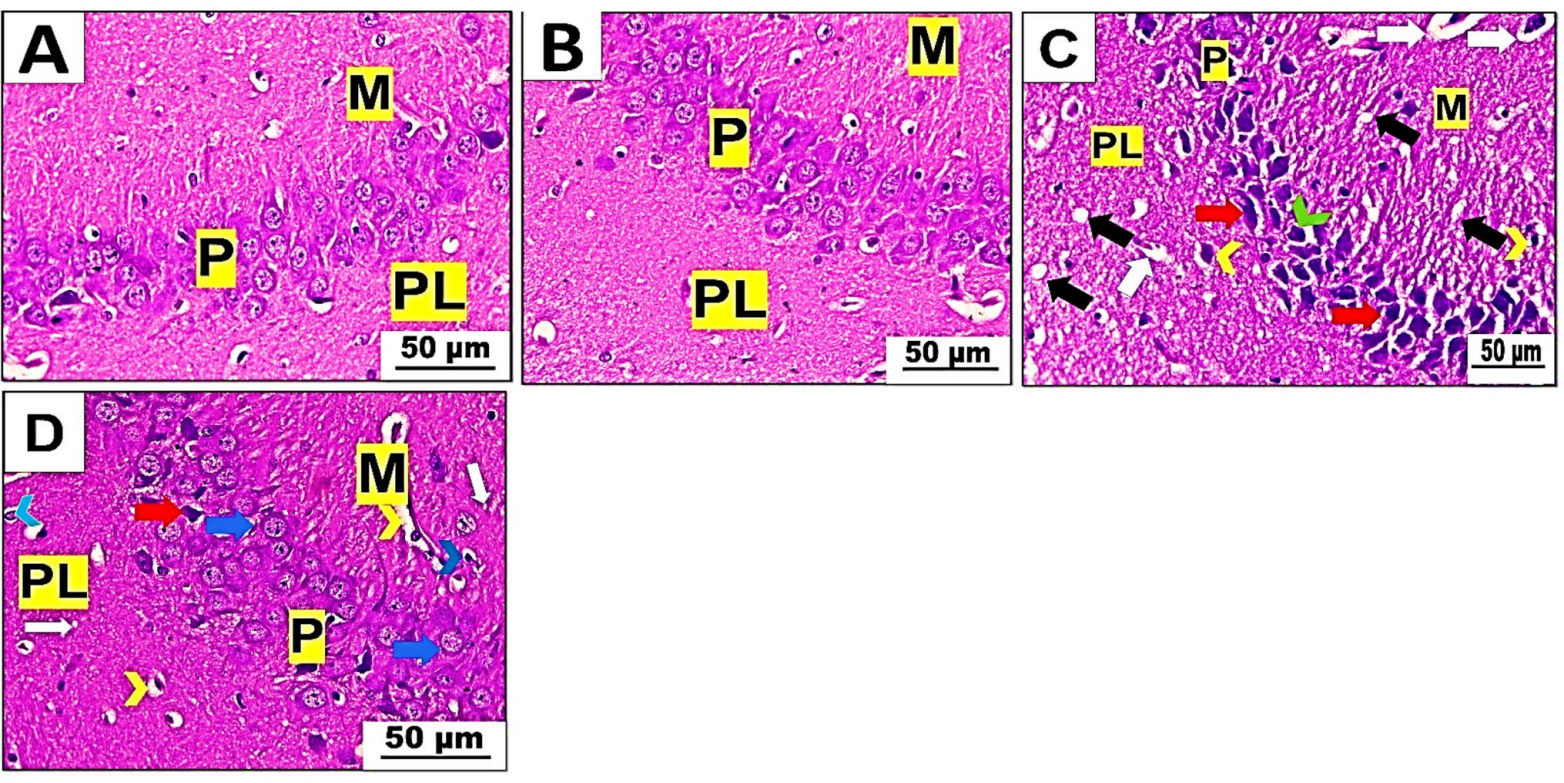
**A**–**D** Photomicrographs of hippocampal sections of Swiss albino mice (*n* = 5 mice/group). H&E stain (× 400). **A** Control group and **B** betaine-administered group demonstrating standard structure and arrangement of molecular cell layer (M), pyramidal cell layer (P), and pleomorphic cell layer (PL). **C** PET-NPs-exposed group exhibiting vacuolations in the neuropil (black arrow), perivascular spaces (white arrow), and pericellular spaces (yellow arrowhead) in both M and PL, and noticeable distorted and shrunken pyramidal cells with pyknotic nuclei (red arrow) and pericellular spaces (green arrowhead) in the P. **D** Betaine-co-treated group showing decreasing pericellular spaces (blue arrowhead), perivascular spaces (yellow arrowhead), and neuropil vacuolation (white arrow) in both M and PL, the most typical structure of the pyramidal cells with vesicular nuclei (blue arrows) but a few neurons still degenerated (red arrow)

The hippocampal slices from mice that were not given betaine (group I) and those that were (group II) showed that the molecular, pyramidal, and polymorphic cell layers were organized in a usual way. Both the molecular and polymorphic cell layers displayed normal-appearing neurons and neuroglia cells. The pyramidal cell layer had many triangular neurons arranged closely together, and their nuclei were spherical and vesicular (Fig. 8A, B). On the other hand, hippocampal sections from mice given PET-NaPs showed spongiosis in the neuropil and spaces around blood vessels and cells in both the molecular and polymorphic cell layers. Additionally, there were severely damaged pyramidal cells that appeared more diminutive in size, with condensed nuclei, and surrounded by spaces near the cells (Fig. 8C). In contrast, the injection of betaine combined with PET-NPs (group IV) effectively protected hippocampal neurons. The molecular and polymorphic cell layers showed a decrease in pericellular and perivascular spaces, along with neuropil vacuolation. Additionally, there was a partial restoration of pyramidal cells, which exhibited a nearly standard structure and contained vesicular nuclei (see Fig. 8D).

#### Immunohistochemical analysis of GFAP

**Fig. 9:**
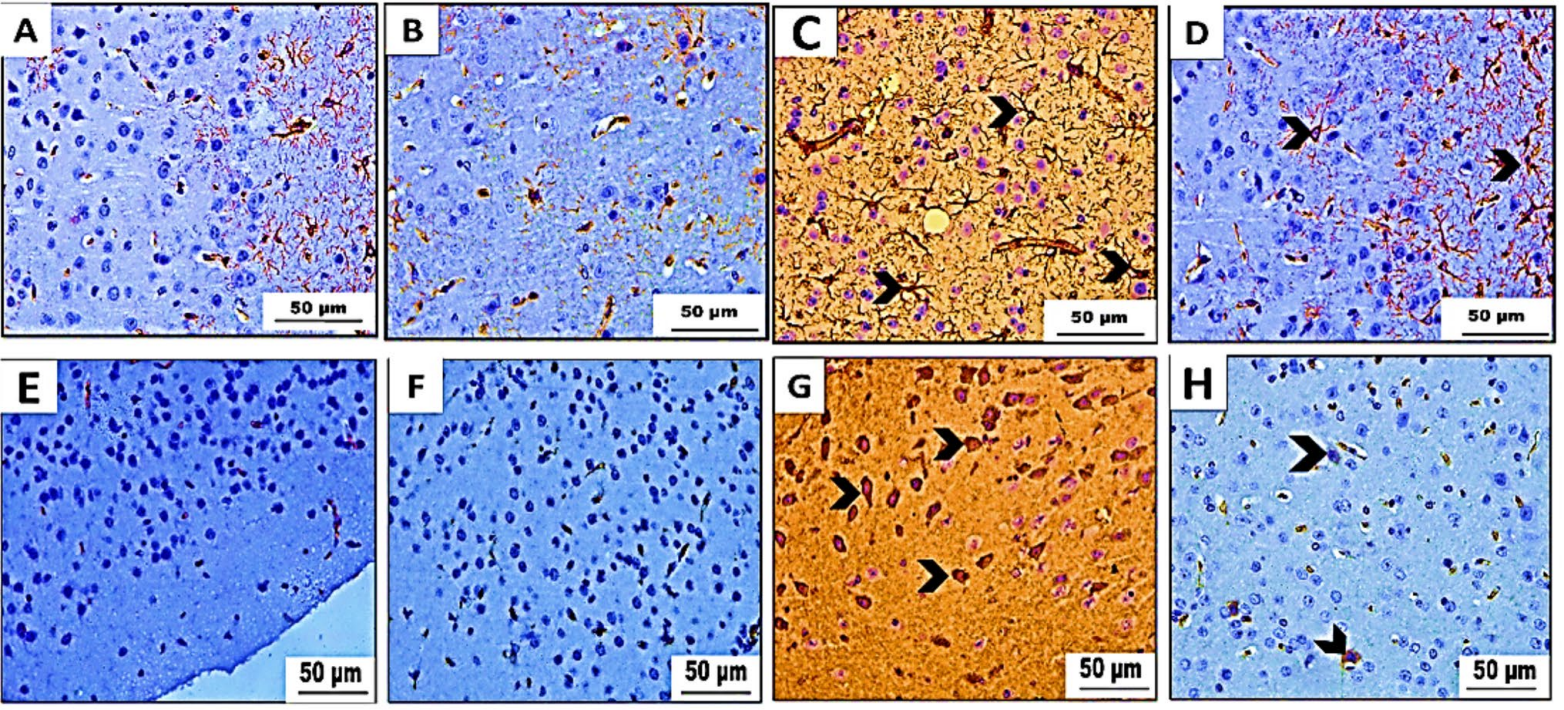
**A**–**D** The results of our experiment. We immunohistochemically analyze GFAP and **E**, **F** IL-1β-stained cerebral cortex sections of Swiss albino mice (*n* = 5 mice/group) (× 400). **A**, **E** Control group (I) and **B**, **F** betaine-treated group (II) showing mild positive (+) GFAP and negative (−) IL-1β immunoreactivity. **C**, **G** PET-NaPs-exposed group (III) showing strong positive (+++) GFAP and strong IL-1β immunoreactivity (arrowhead). **D**, **H** Betaine-co-treated group (IV) showing moderate (++) GFAP immune reaction and weak IL-1β immunoreactivity (arrowhead)

**Fig. 10:**
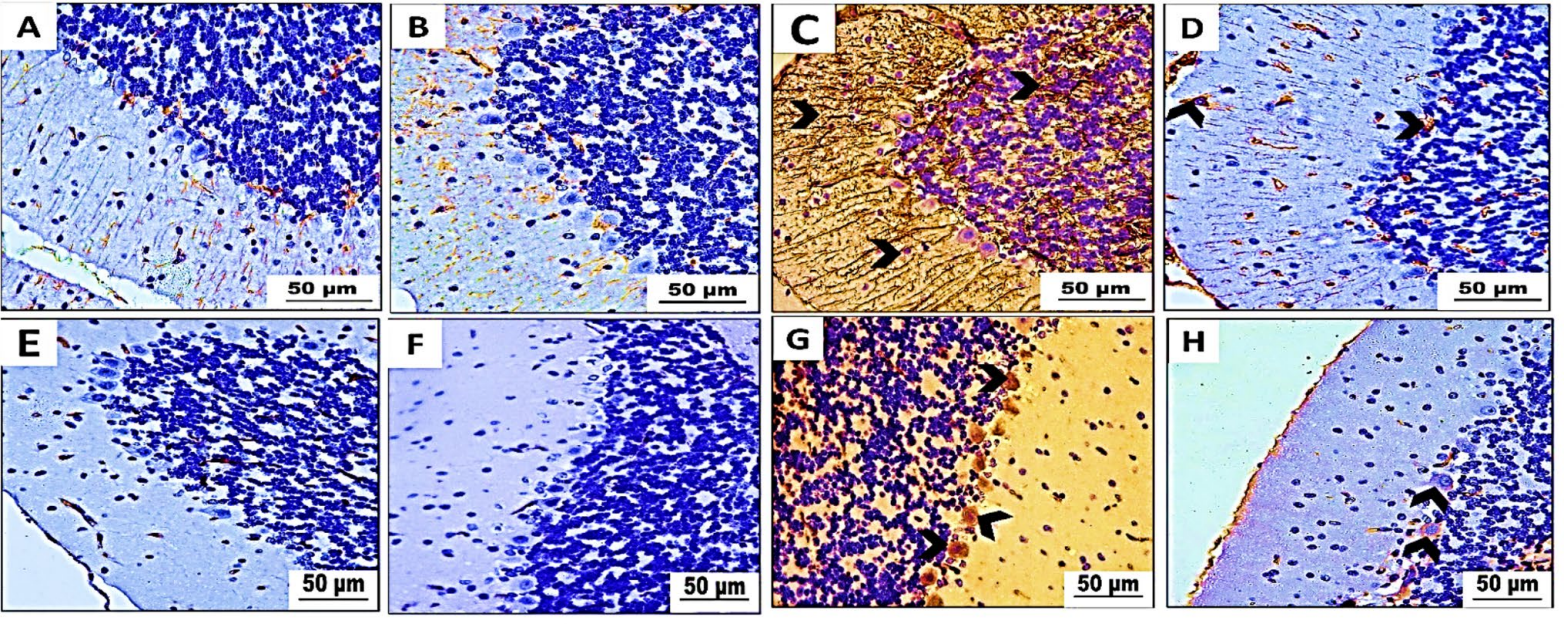
Immunohistochemical analysis results of **A**–**D** GFAP and **E**–**H** IL-1β-stained cerebellar cortex sections of Swiss albino mice (*n* = 5 mice/group) (× 400). **A**, **E** Control group and **B**, **F** betaine-administered group displaying mild positive (+) GFAP immune reaction and negative IL-1β immunoreactivity. **C**, **G** PET-NaPs-exposed group showing strong positive (+++) GFAP immunoreactivity and strong IL-1β immunoreactivity (arrowhead). **D**, **H** Betaine-co-treated group exhibiting moderate (++) GFAP immune expression and weak IL-1β immunoreactivity (arrowhead)

**Fig. 11:**
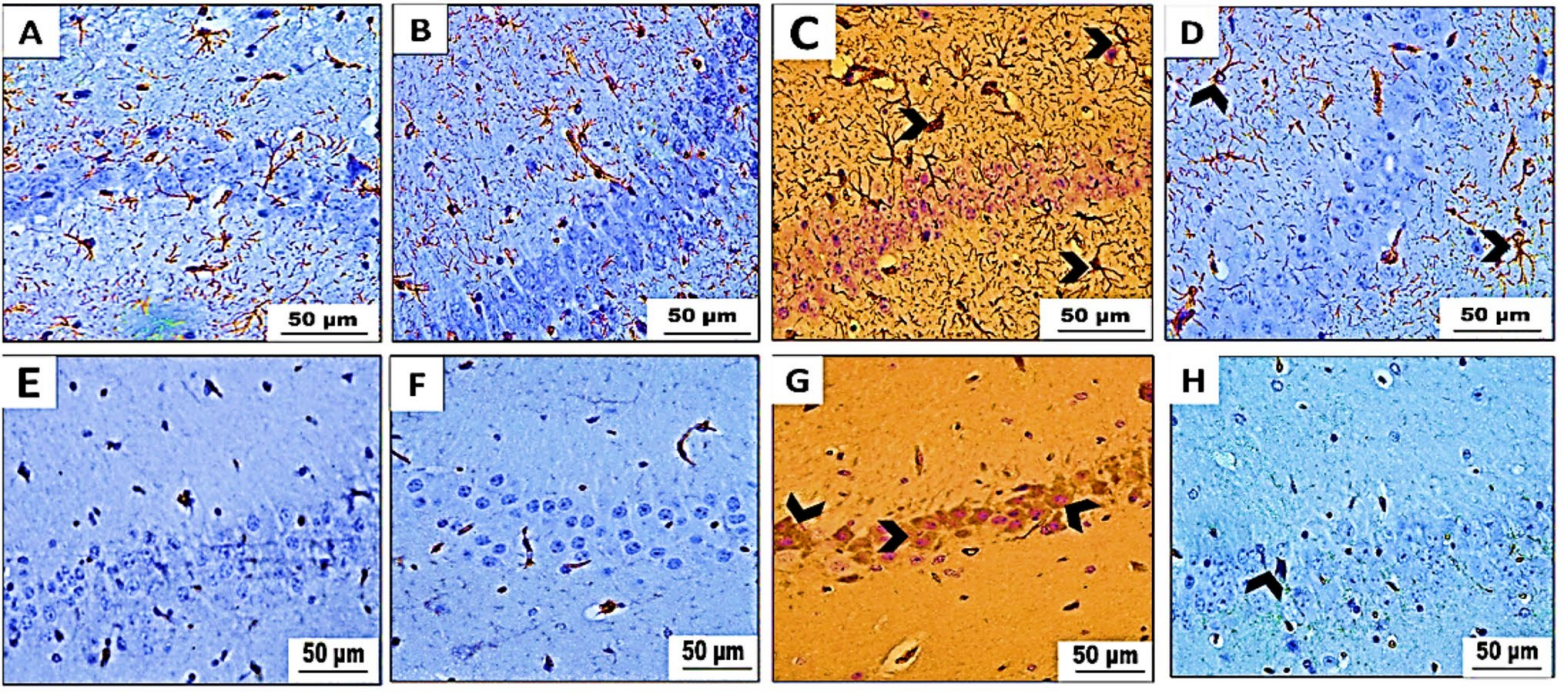
Immunohistochemical analysis results of GFAP and **E**–**H** IL-1β-stained hippocampal brain tissue obtained from Swiss albino mice (*n* = 5 mice/group) (× 400). **A**, **E** Control group and **B**, **F** betaine-administered group revealing mild positive (+) GFAP immune reaction and negative IL-1β immunoreactivity. **C**, **G** PET-NPs-exposed group showing strong positive (+++) GFAP immunoreactivity and strong IL-1β immunoreactivity (arrowhead). **D**, **H** Betaine-co-treated group exhibiting moderate (++) GFAP immune expression and weak IL-1β immunoreactivity (arrowhead)

We conducted an immunohistochemical examination on slices of the cerebrum, cerebellum, and hippocampus from two groups of mice: the control group (group I) and the betaine-administered group (group II). The analysis showed a slight positive (+) immunoreactivity of GFAP in certain areas. Animals in group III were only given PET-NaPs. Sections from these animals showed strong and widespread (+++) GFAP immunological reactivity in the bodies and processes of fibrillary astrocytes. In many places, sections from mice given betaine (group IV) showed a moderate (++) level of immunological expression of GFAP, as shown in Figs. 9, 10, and 11.

##### Immunohistochemical analysis of IL-1β 

Immunohistochemical investigations showed that the brain sections (cerebrum, cerebellum, and hippocampus) obtained from both control mice (group I) and animals subjected to betaine (group II) did not exhibit any IL-1β reactivity. However, brain slices taken from mice treated with PET-NPs (group III) had intense staining that indicated the presence of IL-1β. On the other hand, brain slices from mice in group IV that received both betaine treatments showed a slight immune response for IL-1β, as seen in Figs. 9, 10, and 11.

**Fig. 12:**
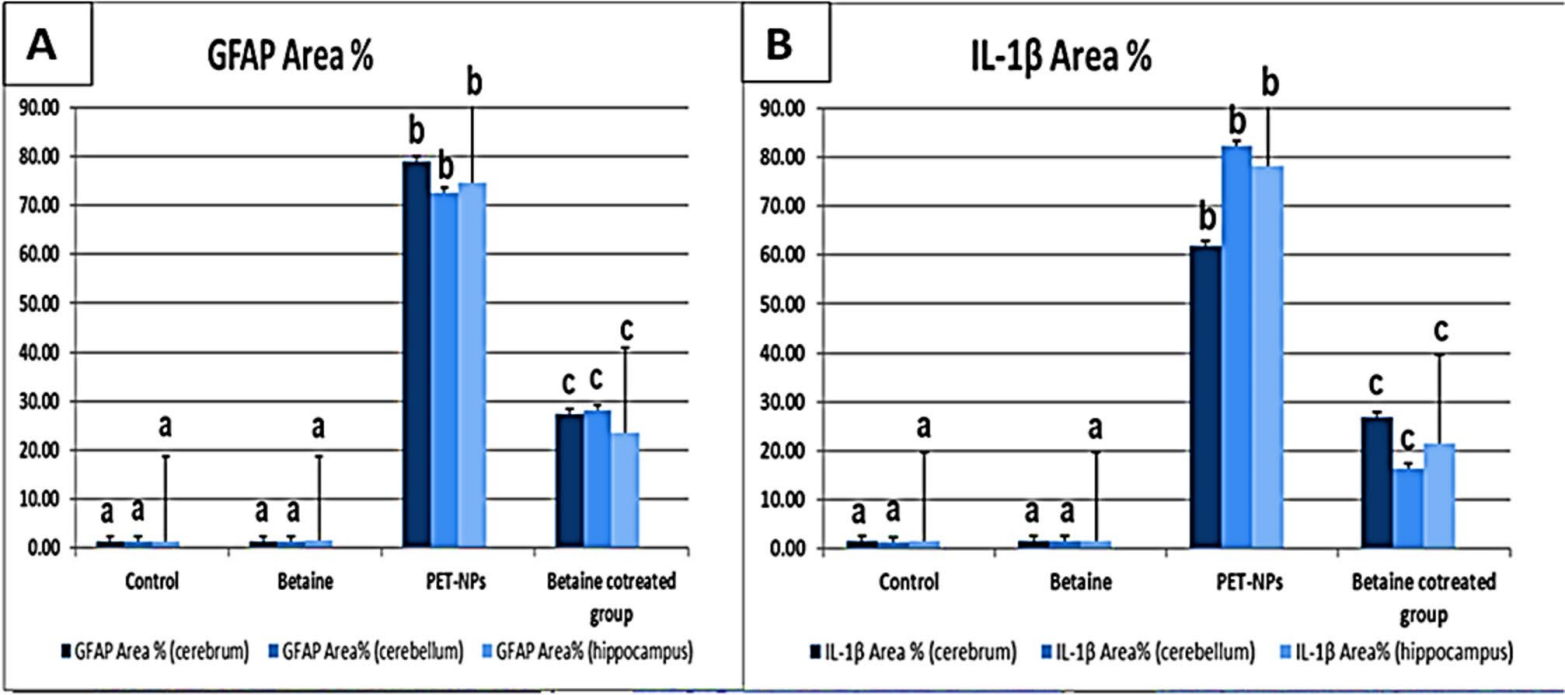
A photomicrograph showing **A** GFAP area% and **B** IL-1β within the cerebrum, cerebellum, and hippocampus in different groups of mice (*n* = 5 mice/group). Data are presented as mean ± SEM. Groups having different letters are significantly different from each other at *P* < 0.001. Groups with similar letters are non-significantly different at *P* < 0.001

The quantification of GFAP and IL-1β immune expression is shown in Fig. 12.

The histopathological analysis of the injuries in the different test groups confirmed that betaine protected neurons from the harmful effects of PET-NPs, as shown in Table 1.

**Table 1:** Cerebral, cerebellar, and hippocampal tissue sections from various experimental groups were evaluated for histopathological lesions

Histopathological scoring	Control group	Betaine group	PET-NaPs-exposed group	Betaine-co-treated group
Neuronal degeneration	0 ± 0^a^	0 ± 0^a^	3 ± 1^b^	1 ± 0^ab^
Dilated perivascular space	0 ± 0^a^	0 ± 0^a^	3 ± 1^b^	1 ± 0^ab^
Spongiosis	0 ± 0^a^	0 ± 0^a^	3 ± 1^b^	1 ± 1^ab^
Hemorrhage	0 ± 0^a^	0 ± 0^a^	3 ± 1^b^	0 ± 1^ab^

The data are presented as median ± interquartile range for six mice in each group

^a^*P* < 0.05 compared to the PET-NaPs group

^b^*P* < 0.05 compared to the control group

## Discussion

Despite the long history of plastic utilization and its widespread in the environment, limited data about NP toxicity in mammals is available. Thus, the current study aimed to evaluate the toxic impact of PET-NaPs on mice’s brain tissue following oral ingestion. It also evaluated the protective impact of betaine against PET-NaPs-induced neurotoxicity. Recent studies have demonstrated that NPs can cross the BBB (Grodzicki et al. 2021). Oxidative stress and inflammation are important in the pathophysiology of NP intoxication (Ahmed et al. 2022). As a result, the current study examined various oxidative stress biomarkers and the mRNA relative expression of some proinflammatory cytokines. The primary mechanism for NP toxicity is believed to be oxidative stress and ROS generation (Ahmed et al. 2022; Yasin et al. 2022).

Our findings indicated that oral ingestion of PET-NaPs resulted in a significant increase in MDA levels and a decrease in GSH content. GSH is an endogenous nonenzymatic cell antioxidant molecule that scavenges and neutralizes ROS. ROS accumulation as a result of endogenous antioxidant inhibition causes damage to biomolecules such as DNA, proteins, and lipid peroxidation, leading to the formation of MDA (Su et al. 2019). MDA is a reliable indicator for lipid peroxidation. The increased levels of MDA and reduced GSH found in the present study point to oxidative stress induction, which can contribute to PET-NaPs-induced brain damage. NaPs may cause cells to make too many ROS, which may lead to changes in metabolic processes like oxidative phosphorylation, the tricarboxylic acid (TCA) cycle, the breakdown of fatty acids, and the metabolism of amino acids (Ahmad et al. 2019; Wang et al. 2023). Additional evidence of brain injury comes from the downregulation of AChE that PET-NaPs produced in our study, which provides additional evidence of brain injury. AChE is responsible for the enzymatic transformation of acetylcholine (ACh) into acetic acid and choline (Wang et al. 2025). ACh is a vital neurotransmitter crucial to memory, learning, and attention (Sun et al. 2015). ACh accumulates due to oxidative stress at cholinergic synapses in the central nervous system and neuromuscular junctions. This accumulation of ACh can result in neuronal dysfunctions, such as increased cholinergic activity in the neuromuscular system (Al Olayan et al. 2020).

PET-NaPs triggered neurodegeneration in the current study, as evidenced by neuropil vacuolation (spongiosis). Additionally, we observed pericellular and perivascular spaces in three brain regions, consistent with the findings of Elnoury et al. (2013), Wang et al. (2022), Abdelhameed et al. (2023), and Yousef et al. (2019). After treating cells with iron oxide and silver nanoparticles, we saw changes like pericellular edema, blood vessel dilation, and vascular damage, resulting in fluid accumulation in the affected area. Furthermore, Scott et al. (2008) proposed that the enlargement of neuronal processes and presynaptic nerve terminals leads to the formation of vacuoles in the neuropil. Galal et al. (2019) suggest that damage to the cytoskeleton can cause neuronal atrophy and process retraction, leading to the vacuolation of the neuropil. Furthermore, loss of neuronal arrangement in cerebral layers also results in distorted, degenerated, and shrunken neurons with pyknotic nuclei. In particular, Xu et al. (2021) found abnormal neuronal layering and deformed neurons in the cerebral cortex of mice exposed to PS-NaPs, which were identified by nuclear pyknosis.

The study also found that the cerebellum had subpial hemorrhage, nuclear pyknosis in the molecular cell layer, deformed and shrunken Purkinje cells with pyknotic nuclei, and the loss of several Purkinje cells with vacuolar spaces in the Purkinje cell layer. Aside from that, the thickness of the granular cell layer appeared to decrease. These findings were in harmony with Xu et al. (2015), Bashir et al. (2021), and Yin et al. (2022). Furthermore, in this study, the pyramidal cells of the hippocampus in Swiss albino mice exposed to PET-NPs appeared distorted and shrunken, with pyknotic nuclei. NPs cause oxidative stress in hippocampal neurons, adversely affecting cellular constituents such as proteins and cell membranes (Beal 1996; Flynn and Melov 2013).

Yang et al. (2023) suggest that NPs generate neurotoxicity through a potential mechanism of neuroinflammation. The current findings demonstrate a significant increase in the expression of COX-2 and IL-1 following oral ingestion of PET-NaPs. The *COX-2* gene is a potent biomarker for tissue inflammation, as Galal et al. (2014) demonstrated. COX-2 is an enzyme that triggers the first stage of generating prostanoids associated with inflammatory and immunosuppressive diseases (Liu et al. 2015). IL-1β is a prototypic proinflammatory cytokine that has pleiotropic effects on many cells and is important in acute and chronic inflammatory and autoimmune diseases. Overproduction of IL-1β is linked to pathophysiological alterations in diseases such as rheumatoid arthritis, neuropathic pain, inflammatory bowel disease, osteoarthritis, vascular disease, multiple sclerosis, and Alzheimer’s disease (Dinarello 1996, 2004; Braddock and Quinn 2004). IL-1β can be produced by glial cells such as Schwann cells, microglia, and astrocytes (Clark et al. 2006; Guo et al. 2007; Thacker et al. 2007). Oxidative stress-induced neuroinflammation is related to ROS overproduction, which could induce neuronal damage through lipid peroxidation of membrane phospholipids and degradation of cytoskeletal proteins (Lan et al. 2024). This results in the attraction of many microglial cells, which engulf the necrotic neurons. Microglial cells also secrete and activate proinflammatory cytokines such as IL (Hassanen et al. 2019). The secretion of IL-1 results in the subsequent induction of other proinflammatory genes, such as *COX-2* (Wang et al. 2023). Moreover, we demonstrated overexpression of IL-1 in PET-NaPs-exposed mice compared to control mice, which was in harmony with the findings of Mitra et al. (2011) and Guo et al. (2013).

When brain tissue damage occurs, the cytoskeleton of cells increases the protein GFAP (Panickar and Norenberg 2005). It shows early biological effects connected to changes in the shape and movement of astrocytes, cell communication, synaptic transmission, and the function of the BBB (Pierozan et al. 2012). In contrast to the control group, the mice given PET-NaPs had higher levels of GFAP in their cerebral cortex, cerebellar cortex, and hippocampal areas. This shows that the PET-NaPs stimulated astrocytes. These findings support the findings of Abdelhameed et al. (2023).

Betaine is a promising neuroprotective agent against several neurological disorders (Rahmani et al. 2019; Arumugam et al. 2021; Li et al. 2022). The neuroprotective effect of betaine could be associated with its antioxidant and anti-inflammatory effects (Medici et al. 2014; Zhang et al. 2016; Yang et al. 2017; Arumugam et al. 2021; Hui et al. 2024). Our findings confirm orally administered betaine’s antioxidant properties and anti-inflammatory effects by increasing GSH content, reducing MDA levels, upregulating AChE, and downregulating *COX-2* and *IL-1* gene expression in brain tissue. Researchers have already found that treating cells with betaine lowered oxidative stress by raising the levels of GSH and antioxidant enzymes like glutathione peroxidase 4 (GPx4) and superoxide dismutase (SOD) (Rahmani et al. 2019; Veskovic et al. 2019; Arumugam et al. 2021). Oral betaine treatment also prevents tissue cysteine and GSH depletion because it is associated with converting homocysteine into methionine, which increases the supply of the methyl group required for GSH synthesis (Jung et al. 2013). Increasing antioxidant levels makes betaine more effective at getting rid of ROS, which may be linked to its ability to reduce inflammation (Jung et al. 2013; Arumugam et al. 2021). Betaine’s ability to reduce the production of C-reactive protein (CRP), IL-6, tumor necrosis factor-alpha (TNF-α), and IL-1 also contributes to its anti-inflammatory effect (Veskovic et al. 2019; Ilyas et al. 2022). Hashim et al. (2024) proved that the betaine-co-treated group alleviated the overexpression of IL-1β.

Microscopic analysis of brain tissues from mice exposed to betaine with PET-NPs revealed a partial restoration of the histological structure in the cerebellar cortex, cerebral cortex, and hippocampus. Additionally, there was a notable decrease in the pericellular and perivascular spaces and the neuropil vacuolations across all brain regions. Moreover, this treatment resulted in a reduction in neuronal degeneration. These observations agreed with Hashim et al. (2024). Furthermore, Hashim et al. (2024) noted a significant decrease in GFAP immune expression in mice co-treated with betaine.

## Conclusion

The present investigation revealed that the administration of PET-NaPs causes significant histological alterations and oxidative stress-induced harm in mice’s cerebral tissue. The generation of an excessive amount of ROS leads to elevated lipid peroxidation and the reduction of antioxidant enzymes, accomplishing this. In addition, PET-NaPs increase the expression of specific inflammation-related genes, such as *COX-2*, *IL-1β*, and *GFAP*, while decreasing the expression of *AChE*. Conversely, simultaneous administration of betaine effectively mitigates all neurotoxicological diseases and amplifies the oxidative damage that PET-NaPs instigate. We can attribute the observed results to betaine’s potent antioxidant and anti-inflammatory characteristics. Our research indicates that betaine has promise as a neuroprotective drug for preventing neurotoxicity generated by PET-NaP.

## Recommendation and limitation

Our research suggests a potential solution to the harmful effects of PET-NPs. If the use of PET-NaPs is unavoidable, betaine could be applied as a protective measure for individuals and animals at higher risk. Future research should explore this possibility further and develop strategies to counteract the impact of PET-NaPs on livestock and human health. We propose betaine as a promising neuroprotective agent that could mitigate the detrimental effects of PET-NaPs.

## Data Availability

All source data for this work (or generated in this study) are available upon reasonable request.

## Abbreviations

*ACh*: Acetylcholine
*AChE*: Acetylcholinesterase
*BBB*: Blood–brain barrier
*CNS*: Central nervous system
*COX-2*: Cyclooxygenase-2
*DLS*: Dynamic light scattering
*GFAP*: Glial fibrillar acid protein
*GSH*: Glutathione
*IL-1β*: Interleukin-1 beta
*LPS*: Lipopolysaccharide
*MDA*: Malondialdehyde
*MPs*: Microplastics
*NBF*: Neutral-buffered formalin
*NaPs*: Nanoplastics
*PET*: Polyethylene terephthalate
*PET-NaPs*: Polyethylene terephthalate nanoplastics
*ROS*: Reactive oxygen species
*SE*: Standard error
*UV*: Ultraviolet
